# Basic values as a motivational framework relating individual values with acculturation strategies among Arab immigrants and refugees across different settlement contexts

**DOI:** 10.3389/fpsyg.2023.1094193

**Published:** 2023-06-05

**Authors:** Hisham M. Abu-Rayya, John W. Berry, Zarina Lepshokova, Momin Alnunu, Dmitry Grigoryev

**Affiliations:** ^1^School of Social Work, University of Haifa, Haifa, Israel; ^2^School of Psychology and Public Health, La Trobe University, Melbourne, VIC, Australia; ^3^Department of Psychology, Queen’s University, Kingston, ON, Canada; ^4^Center for Sociocultural Research, HSE University, Moscow, Russia; ^5^Psychology Program, Doha Institute for Graduate Studies, Doha, Qatar

**Keywords:** acculturation strategies, individual values, immigrants, refugees, adaptation

## Abstract

There is a lack of systematic acculturation research on the motivations underpinning the behavior of migrants, which could explain how they acculturate and adapt to their new country of residence. This paper examines the link between values, using the Schwartz Theory of Basic Human Values, and acculturation strategies among Arab immigrant and refugee groups across different settlement contexts. The results of Study 1 (Arab immigrants; *N* = 456) showed, as hypothesized, positive links between strategies and values: the integration strategy with conservation, social focus, self-protection, and self-transcendence values; assimilation with openness to change, personal focus, and growth values; and separation with conservation, social focus, and self-protection. These findings were generally repeated in Study 2 (Syrian refugees; *N* = 415) except that integration was not associated with self-transcendence and that assimilation was positively linked to self-enhancement instead of openness to change. Our analyses indicated that acculturation preferences are mainly related to motivational values, rather than to different settlement contexts in both samples; however, assimilation seems to be more associated to context than values among the refugee sample. Implications of the findings to the acculturation literature are discussed.

## Introduction

The field of cross-cultural psychology has evolved over the past 50 years. Initially, studies of perception, cognition and personality were dominant interests (Berry et al., 2022). More recently, the analysis of citations by Gabrenya and Glazer (2022) showed there are currently three clusters of interests: values, acculturation, and the self. The first is represented by researchers such as Hofstede, Schwartz and Triandis; the second by Berry, Ward and Phinney; and the third by Markus, Kitayama and Kagitcibasi. Within these clusters, there are researchers who are co-citing each other’s work; however, the clusters are distinct, with less co-citing between them. This suggests that there may be limited conceptual and empirical relationships between them. This minimal relationship is surprising, given that we are all interested in how culture and behavior are connected. The link between the study of values and acculturation is of potential interest, both from a scientific and practical point of view. Do personal values promote certain ways of acculturating?

The concept of *basic values* refers to universal human needs (biological needs, the needs of coherent social interaction and the demands of group life) as conscious goals. The main content aspect that separates values from each other is the type of motivation in which they are reflected. Therefore, individual values can be grouped into sets of values (types of motivation) that share a common goal. Schwartz (1992) grouped individual values into ten different basic types of human motivation, which he understood as the main blocks of values (a total of 10 blocks of values were identified—types of motivation). They determine the direction of both specific actions of a person and all his life activity.

The concept of *acculturation* refers to the cultural and psychological changes that take place in groups and individuals following their inter-cultural contact (Redfield et al., 1936; Sam and Berry, 2016). These changes include many phenomena, such as in food and dress habits, social and personal identities, stress reactions and in preferences regarding the ways in which to acculturate. This later phenomenon has been termed *acculturation strategies* and refers to the various ways in which individuals seek to acculturate as they attempt to adapt to their new societies (Berry, 1980, 1997). They are based on the intersection of attitudes towards two underlying issues: for maintaining their heritage cultures and identities; and for having contact with others in the larger society. The intersection of attitudes toward these two issues leads to four acculturation strategies: integration (preference for both), assimilation or separation (preference for one but not the other) and marginalization (preferences for neither).

The relationship between acculturation strategies and many psychological characteristics has been examined by Schmitz and Schmitz (2022), including personality, psychological adaptation, emotions, coping styles, cognitive styles and emotional intelligence. However, they found little research linking acculturation to values. Despite this finding, there is a plausible relationship between personal values and an individual’s acculturation strategies, since the very conceptualization of these two psychological domains have a large overlap. For example, the acculturation strategies involving the preference for ‘cultural maintenance’ (separation and integration) resemble the values of conservation and self-protection. And the assimilation strategy which involves detachment from the culture of origin for the sake of participation in the larger society resembles the meaning of the values of openness and growth.

This paper examines these possible relationships between values and acculturation strategies conceptually and empirically with an example from a study of samples of Arab immigrants and refugees. It asks the question: *Why do immigrants and refugees give preference to one or another acculturation strategy in the process of intercultural adaptation?* Answering this question is important due to the consequences the acculturation process has for the adaptation of immigrants. As articulated by the *Integration Hypothesis* (Berry, 1997, 2005). For instance, immigrants who are doubly engaged in both their heritage culture and in the larger national society (i.e., who use the integration strategy) have better adaptation than those who adopt another strategy. This assertion has received considerable empirical research support across receiving countries and acculturating groups (e.g., Nguyen and Benet-Martínez, 2013; Safa and Umaña-Taylor, 2021; Stogianni et al., 2021; Berry et al., 2022; Abu-Rayya et al., 2023).

For many years, researchers have sought to clarify the various factors underlying acculturation strategies and adaptation in the hope to assist in a better integration of immigrants. In this regard, Berry (1992) distinguished between factors that existed prior to acculturation and those that arose during acculturation. In the first set are some characteristics of the society of origin, such as the socio-political, economic, and demographics, and the experiences of pre-immigration traumatic events (e.g., Buchanan et al., 2017; Fathi et al., 2018). Also in this pre-existing set are the psychological characteristics that are brought to the acculturation arena by individuals, such as their identities, attitudes and values. Factors that arose during the settlement period that might facilitate or hamper immigrants’ acculturation and adaptation include the multicultural ideology and support from the receiving society (e.g., Berry et al., 2022), perceived discrimination and acceptance (e.g., Lindert et al., 2008; Buchanan et al., 2018), perceived identity incompatibility (e.g., Lepshokova et al., 2018), resilience, and self-perceived cultural competence (e.g., Safdar et al., 2012), and intercultural/social–emotional competence and intentions to return to homeland (e.g., Fathi et al., 2018). Further research has broadened the focus to include immigrants’ other personal psychological characteristics such cognitive styles, emotional intelligence, personality traits, and multicultural personality (Schmitz and Schmitz, 2022).

Less attention, however, has been paid to the role that individual values play in the acculturation of immigrants and their adaptation (e.g., Roccas et al., 2000; Güngör, 2007; Sapienza et al., 2010; Recker et al., 2018; Vishkin et al., 2021; Hanel et al., 2022). We consider that individual values are important features of individuals that exist prior to acculturation, which motivates us to examine them in relation to their acculturation strategies. Individuals bring their values with them to the acculturation process and although values may change with acculturation, they are basic elements in the psychological make-up of individuals, and are thus likely to impact their acculturation preferences. According to Schwartz’s (2012) *Theory of Basic Individual Values*, individual values represent subjective beliefs that an individual holds which are associated with affect, serve as evaluative standards, prioritized according to their relative importance to the individual, define goals that motivate and guide actions, and transcend specific situations. Serving as guiding principles in one’s life and motivating preferences, actions, attitudes, and behaviors, values are as such likely to shape immigrants’ inclination towards a particular acculturation strategy and guide the process whereby the means and activities to actualize their preferred strategy are initiated and sustained. This side of acculturation, which involves agency and motivation, is often overlooked. Motivation as a core dynamic process at the individual level (Grigoryev and Berry, 2022) and agency are related to critical aspects of the acculturation process, such as stress and coping, culture learning, as well as identity and intergroup relations (see Gezentsvey and Ward, 2008). We, thus, believe that careful work that examines the role of individual values in the acculturation process will be useful to the acculturation literature in both theoretical and practical terms. The present study attempts to contribute to the growing, yet scarce, literature in this area of inquiry.

### Motivational frameworks of acculturation

Researchers have sought to clarify the personal motivations underpinning the behaviors of immigrants and their acculturation using one of three motivational theoretical frameworks: (1) a *Dual Process Motivational Model* (e.g., Recker et al., 2018), (2) *Theories of Goal Constructs* that distinguish between motivations and means (e.g., Vishkin et al., 2021), and (3) Schwartz’s (1992, 2012) *Theory of Basic Individual Values* (e.g., Roccas et al., 2000; Güngör, 2007; Sapienza et al., 2010).

Recker et al.’s (2018) proposed dual process model differentiates between immigrants’ motivations for heritage culture maintenance (MCM) and motivations for host culture exploration (MCE). Borrowing concepts from the varied, yet inconsistent psychological literature on motives (such as core motives, Fiske, 2008; self-determination theory of Ryan and Deci (2000) that distinguishes between extrinsic and intrinsic motivations; and novelty-seeking versus need for closure (Kruglanski and Webster, 1996), Recker et al. (2018) argued that motives for conservation, maintenance, stability and need for closure, are likely reflected in MCM, whereas motives of openness to change, exploration, plasticity, novelty-seeking, and intrinsic are reflected in MCE. However, their study did not examine the link between these presumably distinct motives *per se* and acculturation strategies; it was just assumed that MCM and MCE reflect those underlying motivations. Besides, the way they conceptualized and measured MCM and MCE resembles common conceptualizations and measures of Berry’s (1997) two-dimensional acculturation model. Moreover, the motives they highlighted compose a cluster of motives aggregated from scattered and disparate theoretical approaches, hindering their conceptual validity when put together. Thus, the suggested MCM and MCE concepts did not generate strong theoretical and practical insights regarding the distinctive personal motives underlying acculturation strategy preferences.

Vishkin et al.’s (2021) utilizes theories of goal constructs and thus proposes a differentiation between motivations underlying acculturation and means to actualize them, which seems also problematic. Driven by theories of goal constructs, which emphasizes the translation of goals into actions, they broke down acculturation strategies into acculturation motivation (i.e., the extent that an immigrant desires to maintain/adopt their heritage/host culture) and acculturation actions (i.e., behaviors such as language and contact) that satisfy the corresponding acculturation motivation. Their empirical data on immigrants to Israel and the United Kingdom supported the relationships between acculturation motivation and actions. However, the way they conceptualized the underlying acculturation motivations is no different from the way acculturation itself is defined in Berry’s (1997) two-dimensional model. Thus, instead of dealing with the question of what motivates immigrants to prefer one acculturation strategy over another, their proposed perspective in practice focuses on the behavioral outcomes of acculturation preferences themselves.

Given the limitations of the theoretical development of Recker et al.’s (2018) and Vishkin et al.’s (2021) approaches to study acculturation motivations, the present study follows a distinct line of classic research (e.g., Sapienza et al., 2010; Hanel et al., 2022) that builds on the strong theoretical and empirical foundations of Schwartz’s (1992, 2012) *Theory of Basic Individual Values* in an attempt to explore the motivations underlying acculturation strategy preferences among immigrants and refugees.

**Figure 1:**
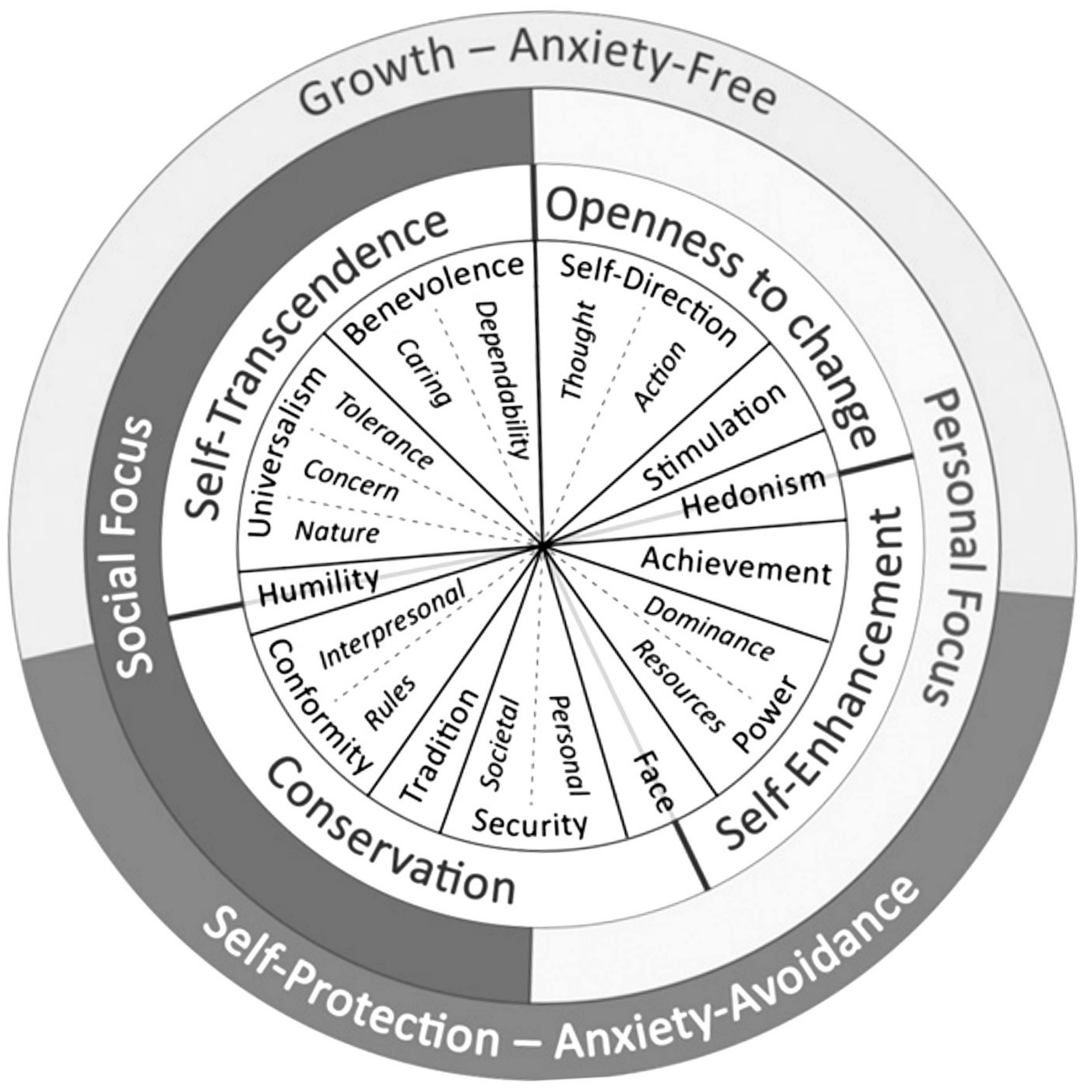
Quasi-circumplex model of human basic values [modified from Cieciuch et al. (2014)].

Ten basic individual values (subdivided later into 19 values) received special attention in Schwartz’s and colleagues’ theorization supported by tremendous empirical evidence across a wide range of countries and cultures (e.g., Schwartz, 1992; Bilsky et al., 2011; Schwartz et al., 2012). These main ten values include Conformity, Tradition, Security, Power, Achievement, Hedonism, Stimulation, Self-Direction, Universalism, and Benevolence, and are distinguished by the type of motivation or goal they express. It is widely accepted to represent these values in a quasi-circumplex motivational continuum, as Figure 1 illustrates. Values that express motivationally incompatible goals are distant from one another, while values that are motivationally compatible sit close to one another in the quasi-circumplex. In addition to the similarity in the structure of the basic values across cultures, there is substantial evidence for the similarity in hierarchies of the values as well. Benevolence, universalism, and self-direction values are best valued, whereas power and stimulation are least important (Schwartz, 2012).

The quasi-circumplex motivational continuum presented in Figure 1 simplifies the compatibility-incompatibility relations between the basic values and the interests that value attainment serves by grouping them at three different levels (presenting four bipolar dimensions): (1) *Conservation* (values emphasizing stability, order, and preservation of traditions) vs. *Openness to change* (values emphasizing change of the status quo, readiness for new experiences/ideas, and independence); and *Self-enhancement* (values emphasizing dominance and success over other and self-interests pursuit) vs. *Self-transcendence* (values emphasizing concern for the welfare and acceptance of others); (2) *Personal focus* (values regulating the expression of personal interests and characteristics) vs. *Social focus* (values regulating social relations and expectations of others); and (3) *Self-protection* (values that are anxiety-based, preventive of loss, protecting against threat) vs., *Growth* (values that are anxiety-free, and promoting goals gain and self-expansion).

The theory of basic individual values have been utilized in acculturation research that sought to find out what values correlate with host society members’ acculturation expectations from immigrants, and also of immigrants’ adoption of a particular acculturation strategy.

Among host society members in Italy, for instance, self-transcendence values were positively associated with integration (and individualism) attitudes towards immigrants and negatively associated with assimilation, separation, and exclusion attitudes, whereas conservation values were negatively associated with integration (and individualism) and positively associated with assimilation, separation, and exclusion strategies (Sapienza et al., 2010). As found by Lepshokova and Vilegjanina (2017), Russian majority mainstream members with self-enhancement values showed more expectation that immigrants to the country should adopt separation or assimilation acculturation strategies and less expectation that they should adopt integration. Self-transcendence values were positively associated with the expectation of integration and negatively associated with the expectation of assimilation (Lepshokova and Vilegjanina, 2017). While less connected with acculturation strategies *per se*, a recent study has also found that self-transcendence positively predicted Singaporeans’ willingness to interact with Chinese immigrants, and conservation values negatively predicted their willingness (See et al., 2020).

Among immigrants, research findings were mixed and inconsistent to some degree. For instance, Roccas et al.’s (2000) study of Russian immigrants to Israel focused just on the conformity value and found no relationship between immigrants’ acculturation strategies and the importance they attributed to conformity. Null relationships between values and acculturation strategies were also reported in a recent study on the acculturation of Arabs in Germany; reasons to immigrate and duration of stay played no role in the relationships (Hanel et al., 2022). Contrary to these null findings, Roccas et al. (2010) found that individuals who generally identified more with their heritage nation endorsed openness to change values less and endorsed conservation values more. In line with these findings, conservation values were associated with the maintenance of heritage culture (and collective mobility), whereas openness and achievement values motivated an orientation towards the receiving society (and individual mobility) among Moroccan and Turkish immigrants in Belgium (Phalet and Swyngedouw, 2004). Among the Russian ethnic minority in the North Caucasian republics self-enhancement values were positively related to the number and frequency of interethnic contacts with ethnic majority (Lepshokova, 2021). Negative relationships between openness to change values and endorsement of the separation strategy were also reported among Russian and Polish minorities in Lithuania, and those who preferred the assimilation strategy, compared to the integration, reported higher degrees of self-enhancement values (Ryabichenko, 2016). Lastly, Zlobina et al. (2008) reported that in a diverse sample of immigrants (Latin American, East European, Arab and African) to Spain, the separation and assimilation styles of acculturation were, respectively, characterized by conservation and openness to change (and mobility) values, and the integration style was characterized by self-promotion values and a greater orientation to achievement.

These studies have provided concepts and findings on which we have designed and carried out the present research. Since separation, and to a lesser extent integration, involves a greater appreciation and preference for ‘cultural maintenance’ by being faithful to the interests and traditions of the culture of origin, we assume that separation and integration acculturation strategies should be motivated by conservation values and values of social-focus and self-protection. However, integration compared to separation involves appreciation for both heritage and host society traditions. Self-transcendence values that emphasize concern and welfare and acceptance of all (individual members and groups) might motivate this preference for integration. The assimilation strategy characterizes immigrants who are less committed to their culture of origin and who are driven by self-interests. Thus, assimilation should be associated with values of openness, self-enhancement, self-focus, and growth.

### Overview of the current study

The current research seeks to examine the motivational nature underlying preferences for different acculturation strategies using Schwartz’s (2012) individual basic values as a theoretical guide. The reviewed research that examined the interconnections between individual values and acculturation may be criticized on a number of grounds that the present study hopes to address.

*First*, to the best of our knowledge no acculturation study has fully examined the higher four bipolar dimensions defined by Schwartz’s (2012) theory (i.e., conservation vs. openness to change, self-enhancement vs. self-transcendence, personal vs. social focus values, and growth vs. self-protection). In fact, some of the scarce research looked only at a particular value such as conformity (e.g., Roccas et al., 2000, 2010), used and measured a value not directly addressed in Schwartz’s (2012) theory (e.g., separateness was used to mean self-direction in Güngör, 2007), or did not apply either the Schwartz’s Value Survey (SVS) or the Portrait Values Questionnaire (PVQ) as classic and established ways of measuring values [e.g., Hanel et al. (2022) applied a short 10 item scale to measure the values]. In contrast, the present study implements the four bipolar dimensions of individual values and the full corresponding SVS measure (Schwartz, 2012) as a theoretical and empirical guide.

*Second*, the available research dealing with values and acculturation is also limited by the way acculturation was measured. Most studies (e.g., Sapienza et al., 2010; Ryabichenko, 2016; Lepshokova and Vilegjanina, 2017; Hanel et al., 2022) have applied a fourfold measure of acculturation where each acculturation strategy was assessed separately through items asking simultaneously about how immigrants relate to their heritage culture and to the larger society. The few other studies (e.g., Güngör, 2007) either analyzed the relationship between values and each of two underlying dimensions of acculturation separately, using bilineal measures of acculturation; or turned the two bilineal measures into the four strategies through dichotomization (e.g., Phalet and Swyngedouw, 2004). These manners of measuring acculturation strategies have their advantages and disadvantages (e.g., Sam and Ward, 2021). A disadvantage of the dichotomization method, for instance, is that dichotomization to produce the four strategies relies on a particular dividing point (e.g., the scalar mid-point) and this might have statistical problems (e.g., Schwartz et al., 2010). The present study advances the literature related to the value-acculturation links by applying a scarcely used method, namely *Euclidean Distance* (Arends-Tóth and van de Vijver, 2006), for measuring acculturation strategies. This method calculates the distance between respondents’ scores on each of the two acculturation dimensions from the optimal score that each of the four acculturation strategies can achieve. Current acculturation research has started to make use of this new method (e.g., Möllering et al., 2014; Abu-Rayya et al., 2023).

*Third*, to our best knowledge only two studies investigated the relationships between values and acculturation among immigrants from Arabic backgrounds (e.g., Phalet and Swyngedouw, 2004; Hanel et al., 2022). Specifically, Hanel et al. (2022) studied Arab refugee males in Germany and Phalet and Swyngedouw (2004) studied Moroccan (Arab) immigrants in Belgium. Both studies suffer from one or two of the abovementioned limitations and Hanel et al.’s (2022) study focused just on male participants. By studying the relationship between values and acculturation strategies among Arab immigrants (*Study* 1) and refugees (*Study* 2), using established methods and theoretical guides, we hope to extend and enrich the cross-cultural psychological literature in this area of inquiry.

*Fourth*, generalization of findings from immigrants to refugees and vice versa requires implementations of the same methods of assessing values and acculturation preferences across immigrant and refugee samples, currently lacking in the literature. There are important context differences between immigrants and refugees (Berry, 2006) including the permanence of staying in the new country, and the voluntary or forced reasons for their moving. Migrants and refugees experience both ‘push’ and ‘pull’ factors in their decisions to migrate. A push factor is a reactive motivation, driven by constraining or exclusionary factors and have generally a negative character. In contrast, a pull factor is a proactive motivation, driven by facilitating or enabling factors and possess generally a positive character. However, refugees generally experience greater push than pull factors than do migrants (Berry, 1992). Research indicates that people’s motivation to immigrate may affect their adaptation in the receiving country (e.g., Winter-Ebmer, 1994; Chirkov et al., 2007) such that migrants with a high ‘need for success’ economically, for instance, outperform those without this motivation, or those who migrated for political reasons (e.g., Winter-Ebmer, 1994). Refugees typically experience more push (negative character) than pull (positive character) factors to move abroad and they experience harsher realities compared to immigrants and their values priorities might be different. The ongoing violent civil war in Syria, which started in 2011, has resulted in millions of refugees who escaped their devastated country. In contrast to this group, Arab citizens from various Arab countries are mainly driven by economic reasons to emigrate. According to the Arab Opinion Index, which is the largest public opinion survey in the Arab world that has been conducted annually since 2011 by the Arab Center for Research & Policy Studies in Qatar, about 22 to 28% of the surveyed people from the Arab region between 2011 and 2022 showed a desire to emigrate, and 58% (in 2022) indicated that looking for better economic opportunities and conditions was the reason for their willingness to emigrate (Arab Centre for Research and Policy Studies, 2022). Comparisons of the values-acculturation relationships between Arab immigrants (*Study 1*) and refugees (*Study 2*), using the same methodology, should shed light on the stability and meaningfulness of the emergent associations despite the differences in the push and pull factors in the background.

*Fifth*, in addition to studying immigrants and refugees who have disparate immigration profiles, we examined the values-acculturation relationships in various settlement contexts (e.g., Arab societies, non-Arab Muslim collectivistic societies, non-Muslim collectivistic Asian societies, and individualistic Western societies). Research highlights that differences in the structure or composition of the acculturation context can have a profound impact on the acculturation experiences of immigrants and refugees (e.g., Buchanan et al., 2018; Titzmann et al., 2020; Berry et al., 2022). In the analyses we conduct to test the relationship between values and acculturation preferences, host society (settlement context) was included as a random part. By adding the host society to the analyses, we could distinguish between host society’s and values’ effects on acculturation preferences, thus inferring about the stability and meaningfulness of the relationships across settlement contexts in each sample.

### Study hypotheses

We hypothesized that:

*H*1: *Conservation* values (that emphasize cohesion, stability, order, preservation of traditions) will be positively associated with separation. Conservation values will also be positively associated with integration, since immigrant and refugee individuals cannot integrate if they lose attachment to their heritage culture.

*H*2: *Openness to change* values (that emphasize change of the status quo, readiness for new experiences/ideas, and independence) will be positively associated with assimilation.

*H*3: *Self-enhancement* values (that emphasize dominance, personal success, and self-interests pursuit) will be positively associated with assimilation.

*H*4: *Self-transcendence* values (that emphasize concern and welfare and acceptance of all, thus both heritage and host society values), will be positively associated with integration.

*H*5: *Personal focus* values (that regulate personal interests’ expression and characteristics) will be positively associated with assimilation.

*H*6: *Social focus* values (that regulate social relations and expectations of others) will be positively associated with separation, and also integration to some extent.

*H*7: *Growth* values (that free the individual from anxieties and promote their personal goals gain and self-expansion) will be positively associated with assimilation.

*H*8: S*elf-protection* values (that are anxiety-driven and their goal is to prevent loss and protect against threat) will be positively associated with separation and integration.

## Study 1: Arab immigrants

The purpose of this study was to examine the relationships between basic individual values and acculturation strategies among Arab immigrants. As noted, immigrants typically voluntarily relocate to a new country to establish a long-term or permanent home. Our immigrants sample comprised Arab immigrants in various settlement contexts so that stability and meaningfulness of the relationships can be inferred across contexts.

### Method

#### Participants

Out of 509 Arab immigrants who entered our online survey, 456 completed it (about 90%). This sample comprised Arab immigrants from various countries (e.g., Algeria, Egypt, Iraq, Jordan, Lebanon, Libya, Morocco, Palestine, Saudi Arabia, etc.). Women made up 41.4% and men 58.6% of the sample; respondents’ age ranged from 18 to 60 YO (*M* = 34.9, *SD* = 9.3); 29.8% were immigrants to Arab Muslim countries (e.g., Egypt, Saudi Arabia), 31.6% were immigrants to non-Arab Muslim countries (e.g., Turkey, Malaysia), 12.3% were immigrants to non-Muslim Asian countries (e.g., China, South Korea), 26.3% were immigrants to Western countries (e.g., United Kingdom and United States). Participants’ mean length of residence in the country of settlement was 9.68 (*SD* = 10.37). The difference in length of residence between Sample 1 (*M* = 9.68) and Sample 2 (*M* = 5.76) was significant (*t*(758) = 6.84, *p* < 0.001) with a medium effect size (Cohen’s *d* = 0.50). Respondents were fairly good educated: 43% had bachelor’s degree, 30% had graduate degree, 19% had post-graduate degree, 5% had post-secondary diploma, 3% had secondary or less qualification. Eight percent indicated having an excellent income, 31% had above average income, 52% had average income, 6% had below average income, and 3% had worse income.

#### Procedure

The sample was recruited by positing a call for participation in the study on various online Arab forums, social media platforms (e.g., LinkedIn and Facebook), and through Arab associations, snowball sampling (by asking participants to share the call with their social networks), and also by posting the call on the first and fourth author’s and their Arab colleagues’ international social networks. Participants responded to an online self-report survey administered on Qualtrics. After giving consent, participants provided information on their country of origin and country of current residence. Only those that their host country of resident was different to their country of origin were targeted and included in this study. The study questionnaires were administered in Arabic as both the acculturation scales and SVS have an Arabic version ready for researchers’ use, and despite respondents’ geographic dispersion over a wide range of Arab and non-Arab countries, Arabic is their common language. The study was conducted in line with the APA Code of Conduct and an ethics approval was granted from the fourth author’s Institution’s Internal Review Board. Data for both Study 1 and Study 2 are available *via* the Open Science Framework.^1^

#### Measures

##### Outcome variable

###### Acculturation Strategy

The study employed the 8-item Brief Acculturation Orientation Scale (BAOS) which is a bilineal measure of acculturation strategies toward the home country and the host country (Demes and Geeraert, 2013). On a 7-point Likert-type scale (1 = ‘*strongly disagree*’ to 7 = ‘*strongly agree*’), participants were asked to rate their agreement with four statements regarding their strategy towards their heritage culture (e.g., “It is important to me to have friends from my home country”); there were also four statements regarding their strategy towards the host society (e.g., “It is important to me to take part in the host country traditions”). The Cronbach alpha was 0.84 for the heritage culture dimension and 0.78 for host society dimension of the acculturation measure. We followed the steps of research that applied the two-dimensional space Euclidian distance formula to calculate participants’ scores on the acculturation strategies (Arends-Tóth and van de Vijver, 2006; Möllering et al., 2014; Abu-Rayya et al., 2023). Scores on both the two acculturation dimensions were used in the calculations. The formula is as follows: $\sqrt{{\left(Q1-P1\right)}^{2}+{\left(Q2-P2\right)}^{2}}$ where (*P*1, *P*2) and (*Q*1, *Q*2) represent the coordinates. The coordinate (*P*1, *P*2) represents the extreme score that an acculturation strategy can achieve. In our study, the highest score for integration is (7,7), which reflects the max score on both acculturation dimensions (7,1) for separation, and (1,7) for assimilation. Euclidean distance scores are then reversed to reflect how close a participant’s score will be to each acculturation strategy. Marginalization showed a very strong negative correlation with integration, *r* = − 0.94, *p* < 0.001. This means that close to 90% of the variation of marginalization scores in our sample is explained by integration scores. Hence, marginalization in our sample seems to reflect people with low score on the integration strategy, rather than a genuine preference for marginalization. This inference is supported by a separate analysis of the distribution of the acculturation strategies showing that just 45 immigrants in our sample scored below the midpoint (i.e., 4) in each of the two acculturation dimensions. Marginalization occurs infrequently here, and thus its viability as an acculturation preference may be questioned, as also noted by Ward and Geeraert (2016). Indeed, some acculturation research based on data-driven approach like latent profile analysis usually did not find any marginalization profiles (e.g., Grigoryev and van de Vijver, 2017). In subsequent analyses of the relationship between values and acculturation, we thus only focused on integration, assimilation, and separation scores.

##### Antecedents

###### Basic individual values

The 10 basic individual values (Power, Achievement, Hedonism, Stimulation, Self-Direction, Universalism, Benevolence, Tradition, Conformity, Security) were measured by the original SVS which includes 57 items (Schwartz, 1992, 2012). Respondents indicated how important each value is for them as a guiding principle in his/her life, such as “___SOCIAL POWER (control over others, dominance)” on a 9-point Likert-type scale (− 1 = ‘*opposed to my values*’, 7 = ‘*of supreme importance*’). Scores for each participants on each value were centered around their mean score (Schwartz, 1992). Scores for the eight higher values of conservation (*α* = 0.73), openness to change (*α* = 0.62), self-enhancement (*α* = 0.52), self-transcendence (*α* = 0.75), growth (*α* = 0.80), self-protection (*α* = 0.72), personal focus (*α* = 0.68), and social focus (*α* = 0.84) were generated and used in the analyses. Cronbach’s α reliability of the values are comparable to those reported in the literature on values. For instance, Schwartz (2021) noted that the average Cronbach’s α reliability of the SVS values is 0.61 with a range from 0.54 to 0.71, and that multidimensional scaling shows adequate discrimination of values.

#### Results

Pearson’s bivariate correlations between individual values and acculturation strategies for Study 1 are displayed in Table 1. The correlations are all in the hypothesized directions of H1–H8, and they ranged from 0.13 (for self-enhancement and assimilation) to 0.30 (for social-focus/self-protection and integration).

**Table 1:** Descriptive statistics and bivariate correlations for study 1 (*N* = 456) / Study 2 (*N* = 415).

	1	2	3	4	5	6	7	8	9	10	11
1. Integration											
2. Assimilation	0.08/0.05										
3. Separation	0.20^*^/0.09	−0.77^*^/−0.79^*^									
4. Openness to change	0.10^*^/0.17^*^	0.22^*^/0.20^*^	−0.10^*^/−0.15^*^								
5. Self-enhancement	0.15^*^/0.28^*^	0.13^*^/0.15^*^	−0.06/−0.10^*^	0.60^*^/0.59^*^							
6. Conservation	0.28^*^/0.42^*^	−0.05/−0.15^*^	0.23^*^/0.24^*^	0.13^*^/0.02	0.20^*^/0.22^*^						
7. Self-transcendence	0.26^*^/0.32^*^	0.07/−0.03	0.08/0.06	0.37^*^/0.31^*^	0.29^*^/0.24^*^	0.64^*^/0.60^*^					
8. Personal focus	0.17^*^/0.29^*^	0.20^*^/0.15^*^	−0.07/−0.10^*^	0.83^*^/0.82^*^	0.90^*^/0.91^*^	0.25^*^/0.22^*^	0.42^*^/0.33^*^				
9. Social focus	0.30^*^/0.41^*^	0.01/−0.10^*^	0.17^*^/0.17^*^	0.28^*^/0.18^*^	0.28^*^/0.26^*^	0.90^*^/0.89^*^	0.91^*^/0.90^*^	0.37^*^/0.31^*^			
10. Growth	0.22^*^/0.33^*^	0.17^*^/0.10^*^	−0.02/−0.05	0.78^*^/0.76^*^	0.61^*^/0.60^*^	0.49^*^/0.43^*^	0.84^*^/0.82^*^	0.80^*^/0.77^*^	0.74^*^/0.70^*^		
11. Self-protection	0.30^*^/0.46^*^	0.01/−0.09	0.17^*^/0.18^*^	0.28^*^/0.17^*^	0.57^*^/0.57^*^	0.90^*^/0.91^*^	0.64^*^/0.56^*^	0.58^*^/0.53^*^	0.84^*^/0.82^*^	0.64^*^/0.55^*^	
*Mean* *^#^*	0.64/0.62	0.50/0.53	0.59/0.55	0.49/0.52	0.51/0.50	0.58/0.50	0.67/0.67	0.51/0.51	0.69/0.64	0.59/0.60	0.56/0.49
*SD*	0.17/0.18	0.14/0.16	0.15/0.17	0.18/0.19	0.17/0.17	0.15/0.16	0.15/0.15	0.17/0.17	0.15/0.16	0.16/0.16	0.16/0.17

* *p* < 0.05.

# Since the response scales of the various measures had a different number of points (i.e., 7 and 9), a rescaling procedure was carried out defining a new scale range from 0.01 to 1, where 0.01 = a minimum level of the measured quality and 1 = a maximum level of the quality.

To test our hypotheses, we conducted a series of three separate Mixed Models. In all these analyses individual values were input in the fixed part of the model, and respondents’ country of origin and their destination country were defined as the random part. In one model conservation, openness to change, self-enhancement, and self-transcendence were put together because they operate at the first level in the basic values theory (Table 2); in the second model personal focus and social focus values were put together because they are defined at the second level in the theory (Table 3), and finally, self-protection and growth were put together as they define the third level in the theory (Table 4). We inspected the data for homoscedasticity, normality of residuals, and multicollinearity. Visual graphs for the various Mixed Models, did not indicate violation of homoscedasticity and normality of residuals, and the predictor variables did not show substantial multicollinearity, with VIFs ranging from 1.04 to 2.01 (Mean = 1.27).

**Table 2:** Level 1 values and acculturation strategies: unstandardized estimates and 95% CI of mixed effects linear regression for study 1 (*N* = 456).

	Acculturation strategies						Integration		Assimilation		Separation	
Fixed part
Individual values						
Openness to change	−0.003	[−0.114, 0.108]	**0.168** ^*^	**[0.073, 0.263]**	−0.080	[−0.180, 0.021]
Self-enhancement	0.093	[−0.019, 0.205]	0.005	[−0.091, 0.100]	−0.029	[−0.131, 0.072]
Conservation	**0.206** ^*^	**[0.076, 0.336]**	**−0.126** ^*^	**[−0.237, −0.015]**	**0.285** ^*^	**[0.168, 0.401]**
Self-transcendence	**0.146** ^*^	**[0.006, 0.286]**	0.068	[−0.052, 0.188]	−0.057	[−0.183, 0.070]
Sociodemographic variables						
Gender (1 = male)	0.015	[−0.017, 0.046]	0.008	[−0.019, 0.036]	−0.010	[−0.038, 0.019]
Age	0.001	[−0.001, 0.002]	−0.001	[−0.002, 0.001]	0.001	[−0.001, 0.003]
Education	**−0.031** ^*^	**[−0.047, −0.014]**	0.005	[−0.010, 0.019]	**−0.015** ^*^	**[−0.030, −0.001]**
Income	0.003	[−0.016, 0.022]	0.001	[−0.016, 0.016]	0.004	[−0.013, 0.021]
Length of residence	**−0.002** ^*^	**[−0.003, −0.001]**	**0.003** ^*^	**[0.002, 0.005]**	**−0.003** ^*^	**[−0.004, −0.001]**
Random part			
*σ* ^2^	0.03	0.02	0.02
τ00 Country of Origin	0.01	0.01	0.01
τ00 Host Country	0.01	0.01	0.01
ICC Country of Origin	0.05	0.01	0.01
ICC Host Country^#^	0.01	0.02	0.01
*N* Country of Origin	18	18	18
*N* Host Country	33	33	33
*R^2^*Marginal/*R^2^*Conditional	0.12/0.16	0.10/0.13	0.10/0.11

Bold figures are significant.

* *p* < 0.05.

# When replacing host country by the grouping host country variable (Arab-Muslim countries, non-Arab Muslim countries, non-Muslim Asian countries, and Western countries), ICC becomes much closer to zero.

**Table 3:** Level 2 values and acculturation strategies: unstandardized estimates and 95% CI of mixed effects linear regression for study 1 (*N* = 456).

	Acculturation strategies						Integration		Assimilation		Separation	
Fixed part
Individual values						
Personal focus	0.093	[−0.005, 0.191]	**0.190** ^*^	**[0.105, 0.275]**	**−0.132** ^*^	**[−0.221, −0.042]**
Social focus	**0.297** ^*^	**[0.191, 0.403]**	−0.070	[−0.161, 0.022]	**0.217** ^*^	**[0.120, 0.313]**
Sociodemographic variables						
Gender (1 = male)	0.014	[−0.017, 0.045]	0.008	[−0.019, 0.036]	−0.011	[−0.039, 0.018]
Age	0.001	[−0.001, 0.002]	−0.001	[−0.003, 0.001]	0.002	[−0.001, 0.003]
Education	**−0.032** ^*^	**[−0.048, −0.015]**	0.004	[−0.010, 0.019]	**−0.016** ^*^	**[−0.031, −0.001]**
Income	0.004	[−0.015, 0.022]	−0.002	[−0.018, 0.014]	0.007	[−0.010, 0.024]
Length of residence	**−0.002** ^*^	**[−0.003, −0.001]**	**0.003** ^*^	**[0.002, 0.005]**	**−0.002** ^*^	**[−0.004, −0.001]**
Random part			
σ^2^	0.03	0.02	0.02
τ00 Country of Origin	0.01	0.01	0.01
τ00 Host Country	0.01	0.01	0.01
ICC Country of Origin	0.06	0.02	0.01
ICC Host Country^#^	0.01	0.02	0.01
*N* Country of Origin	18	18	18
*N* Host Country	33	33	33
*R^2^*Marginal / *R^2^*Conditional	0.12/0.17	0.08/0.13	0.08 /0.10

Bold figures are significant.

* *p* < 0.05.

# When replacing Host Country by the Grouping host country variable (Arab-Muslim countries, non-Arab Muslim countries, non-Muslim Asian countries, and Western countries), ICC becomes much closer to zero.

**Table 4:** Level 3 values and acculturation strategies: unstandardized estimates and 95% CI of mixed effects linear regression for study 1 (*N* = 456).

	Acculturation strategies						Integration		Assimilation		Separation	
Fixed part
Individual values						
Growth	0.091	[−0.033, 0.215]	**0.251** ^*^	**[0.144, 0.357]**	**−0.194** ^*^	**[−0.307, −0.081]**
Self-protection	**0.274** ^*^	**[0.149, 0.399]**	**−0.155** ^*^	**[−0.262, −0.048]**	**0.289** ^*^	**[0.177, 0.402]**
Sociodemographic variables						
Gender (1 = male)	0.013	[−0.018, 0.044]	0.011	[−0.016, 0.039]	−0.014	[−0.043, 0.014]
Age	0.001	[−0.001, 0.002]	−0.001	[−0.003, 0.001]	**0.002** ^*^	**[0.001, 0.003]**
Education	**−0.032** ^*^	**[−0.049, −0.015]**	0.004	[−0.010, 0.019]	**−0.016** ^*^	**[−0.031, −0.001]**
Income	0.002	[−0.017, 0.020]	0.002	[−0.015, 0.018]	0.002	[−0.015, 0.019]
Length of residence	**−0.002** ^*^	**[−0.003, −0.001]**	**0.003** ^*^	**[0.002, 0.005]**	**−0.002** ^*^	**[−0.004, −0.001]**
Random part			
σ^2^	0.03	0.02	0.02
τ00 Country of Origin	0.01	0.01	0.01
τ00 Host Country	0.01	0.01	0.01
ICC Country of Origin	0.05	0.02	0.01
ICC Host Country^#^	0.01	0.02	0.01
*N* Country of Origin	18	18	18
*N* Host Country	33	33	33
*R^2^*Marginal/*R^2^*Conditional	0.12 /0.16	0.09 /0.12	0.09 /0.10

Bold figures are significant.

* *p* < 0.05.

# When replacing Host Country by the Grouping host country variable (Arab-Muslim countries, non-Arab Muslim countries, non-Muslim Asian countries, and Western countries), ICC becomes much closer to zero.

For first level values, only conservation, openness to change, and self-transcendence predicted acculturation strategies after controlling for demographics. Supporting our H1, conservation was positively associated with separation, *B* = 0.29 [*95% CI* = 0.17—0.40], and integration, *B* = 0.21 [*95% CI* = 0.08—0.34], endorsement. Findings provided support also for H2 and H4: openness to change was positively linked to assimilation strategy, *B* = 0.17 [*95% CI* = 0.07—0.26], and self-transcendence was positively associated with integration, *B* = 0.15 [*95% CI* = 0.01—0.29]. As indicated by *R^2^* Marginal estimates, individual values explained between 10 and 12% of the total variance in each strategy. Respondents’ country of origin explained between 1 and 5% of the total variance in each acculturation strategy, whereas host society (acculturation context) explained between 1 and 2%, as the corresponding Intraclass Correlations (ICC) show.

For second level values, the analyses also supported H5 and H6. Personal focus values were positively associated with assimilation, *B* = 0.19 [*95% CI* = 0.11—0.28], and social focus values were positively associated with separation, *B* = 0.22 [*95% CI* = 0.12—0.31], and integration, *B* = 0.30 [*95% CI* = 0.19—0.40]. Values here explained between 8 and 12% of the total variance in each strategy. Respondents’ country of origin and host society (acculturation context) explained 1–6% and 1–2%, respectively, of the total variance in the strategies.

The analysis related to third level values supported H7 and H8. Growth values were positively associated with assimilation, *B* = 0.25 [*95% CI* = 0.14—0.36], and self-protection values were positively associated with separation, *B* = 0.29 [*95% CI* = 0.18—0.40], and integration, *B* = 0.27 [*95% CI* = 0.15—0.40]. As indicated by *R^2^*Marginal estimates, these values explained between 9 and 12% of the total variance in each strategy. Respondents’ country of origin and host society (acculturation context) explained 1–5% and 1–2%, respectively, of the total variance in the strategies.

Readers interested in similar analyses pertaining to the relationship between values and each of the acculturation strategy dimensions (heritage and host society) are referred to Supplementary Tables S1–S3 in the Supplementary material.

#### Discussion

Findings of *Study 1* supported most of our hypothesized positive links between acculturation strategies and individual values. In particular, integration and separation strategies were predicted by conservation, social focus, and self-protection values. A positive link emerged also between self-transcendence values and integration. Assimilation, on the other hand, was positively linked to openness to change, personal focus, and growth values, and contrary to our hypothesis, self-enhancement played no role in the assimilation strategy. The values of conservation and self-protection were negatively associated with assimilation, in contrast to their significant positive relationship with integration and separation. Thus, these values seem not only explaining the motivation for a particular strategy (separation and integration), like the other values, but seem also to explain the discouragement for another (assimilation here). Our analyses indicated that immigrants’ settlement contexts played a minor role in immigrants’ acculturation strategies, compared to individual values. This furthers our confidence in the importance of values in facilitating immigrants’ acculturation strategies. These findings have the potential to support theorization on the motivational underpinning of immigrants’ acculturation strategies. To examine whether the emergent links between values and strategies can be generalized to refugees, we conducted *Study 2*, which involves Syrian refugees who share similar cultural characteristics with Arab immigrants (*Study 1*).

## Study 2: Arab refugees

The purpose of this study was to examine the relationships between basic human values and acculturation strategies among Arab (Syrian) refugees in various national settlement contexts. Refugees typically experience extremely disruptive events in their homelands associated with war and trauma and often resulting in negative psychological consequences such that their migration is typically forced, compared to voluntary migration. Millions of Syrian refugees who escaped their devastated homeland since the war started in 2011 are no exception. Comparing the values-acculturation relationships between the refugee and immigrant samples helps to shed light on the stability and meaningfulness of the relationships across samples, despite disparate backgrounds and conditions.

### Method

#### Participants

Participants for this study were 415 Syrian refugee respondents out of 427 who entered our online survey (about 97%) Of those 415 respondents, 39.5% were women and 60.5% men. Respondents’ age ranged from 18 to 60 YO (*M* = 35.8, *SD* = 9.3), 13.3% of the sample were refugees to Arab Muslim countries (e.g., Egypt, Jordan), 34.5% were refugees to non-Arab Muslim countries (e.g., Turkey, Malaysia), 4.33% were refugees to non-Muslim collectivistic Asian countries (e.g., China, Japan), 48% were refugees to individualistic Western countries (e.g., France, Germany). Participants’ Mean length of residence in the country of settlement was 4.63 (*SD* = 2.97). Participants’ educational level was fairly good: 41% had bachelor’s degree, 24% had graduate degree, 10% had post-graduate degree, 14% had post-secondary diploma, and 11% had secondary or less qualification. The reported income level of the participants was excellent for 3%, above average for 27%, average for 54%, below average for 12%, worse for 4%; less than 1% provided no answer.

#### Procedure

Study procedure was similar to the one in Study 1. In the recruitment process, however, we approached Syrian organizations abroad, online forums, and refugee forums and networks. Ethics approval to conduct the study was granted together with the approval of Study 1 and data collection occurred simultaneously.

#### Measures

We applied the same measures as in Study 1. Reliability of heritage culture dimension of the BAOS measure was Cronbach’s *α* = 0.83, and Cronbach’s *α* = 0.83 for host society dimension. Reliabilities of the eight higher values were generally good: conservation (*α* = 0.79), openness to change (*α* = 0.71), self-enhancement (*α* = 0.59), self-transcendence (*α* = 0.79), growth (*α* = 0.82), self-protection (*α* = 0.79), personal focus (*α* = 0.73), and social focus values (*α* = 0.86). As in Study 1, marginalization showed a very strong negative correlation with integration, *r* = − 0.93, *p* < 0.001, and was excluded from further analyses for the same aforementioned empirical reasons.

#### Results

Preliminary Pearson’s correlational analyses for Study 2 displayed in Table 1 are generally in line with those reported for Study 1. They follow the hypothesized directions reported in H1-H8, and ranged in value from 0.10 (for personal growth and assimilation) to 0.46 (for social-focus and integration).

To test our hypotheses, we conducted a similar set of Mixed Models to Study 1, except that Syrian refugees’ host country only was defined as the random part in the models. Also here, we did not notice a violation of homoscedasticity and normality of residuals, and the predictor variables did not show multicollinearity (VIFs ranged from 1.04 to 2.01; *Mean* = 1.27). For first level values, only conservation and self-enhancement predicted acculturation strategies after controlling for demographics. Supporting our H1, conservation was positively associated with separation, *B* = 0.33 [*95% CI* = 0.20–0.46], and integration, *B* = 0.38 [*95% CI* = 0.25–0.50] strategies. Findings supported H3 of a positive link between self-enhancement and assimilation strategy, *B* = 0.12 [*95% CI* = 0.02–0.23]. As indicated by *R^2^*Marginal estimates, while values explained between 6 and 10% of the total variance in assimilation and separation, respectively, they explained 23% of the total variance in integration. Host society (acculturation context) explained a negligible variance in integration or separation adoption (1%—2%), whereas it explained 14% of assimilation adoption (Table 5).

**Table 5:** Level 1 values and acculturation strategies: unstandardized estimates and 95% CI of mixed effects linear regression for study 2 (*N* = 415).

	Acculturation strategies						Integration		Assimilation		Separation	
Fixed part
Individual values						
Openness to change	0.044	[−0.065, 0.153]	0.103	[−0.002, 0.208]	−0.060	[−0.171, 0.051]
Self-enhancement	**0.169** ^*^	**[0.057, 0.282]**	**0.124** ^*^	**[0.015, 0.233]**	**−0.132** ^*^	**[−0.247, −0.017]**
Conservation	**0.376** ^*^	**[0.252, 0.500]**	**−0.140** ^*^	**[−0.265, −0.016]**	**0.328** ^*^	**[0.200, 0.456]**
Self-transcendence	0.055	[−0.084, 0.193]	−0.015	[−0.150, 0.119]	−0.083	[−0.225, 0.059]
Sociodemographic variables						
Gender (1 = male)	0.012	[−0.020, 0.044]	0.004	[−0.027, 0.035]	−0.006	[−0.039, 0.027]
Age	0.001	[−0.001, 0.003]	0.001	[−0.002, 0.002]	−0.001	[−0.002, 0.002]
Education	−0.010	[−0.025, 0.005]	0.001	[−0.015, 0.014]	−0.001	[−0.005, 0.025]
Income	0.019	[−0.002, 0.039]	0.005	[−0.025, 0.015]	0.008	[−0.013, 0.029]
Length of residence	−0.001	[−0.004, 0.002]	0.002	[−0.001, 0.005]	**−0.003** ^*^	**[−0.006, −0.001]**
Random part			
σ^2^	0.02	0.02	0.03
τ00 Host Country	0.01	0.01	0.01
ICC Host Country^#^	0.01	0.14	0.02
*N* Host Country	34	34	34
*R^2^*Marginal / *R^2^*Conditional	0.23/0.23	0.06/0.19	0.10/0.12

Bold figures are significant.

* *p* < 0.05.

# When replacing Host Country by the Grouping host country variable (Arab-Muslim countries, non-Arab Muslim countries, non-Muslim Asian countries, and Western countries), ICC becomes much closer to zero.

For second level values, the analyses supported H5 and H6, as in Study 1. Personal focus values were positively associated with assimilation, *B* = 0.20 [*95% CI* = 0.11–0.29], and social focus values were positively associated with separation, *B* = 0.24 [*95% CI* = 0.14–0.35], and integration, *B* = 0.39 [*95% CI* = 0.29–0.49]. While values explained 5–7% of the total variance in assimilation and separation, they explained 21% of the integration strategy’s total variance. Again, host society (acculturation context) explained a negligible variance in integration or separation adoption (1–3%), but it explained 15% of total variance in assimilation.

Finally, the analysis related to third level values supported H7 and H8, consistent with Study 1. Growth values were positively associated with assimilation, *B* = 0.19 [*95% CI* = 0.07—0.31], and self-protection values were positively associated with separation, *B* = 0.28 [*95% CI* = 0.17—0.40], and integration, *B* = 0.41 [*95% CI* = 0.30–0.51]. As *R^2^* Marginal estimates implied, these values explained 5–7% of the total variance in assimilation and separation, and 23% of the integration strategy’s total variance. Host society (acculturation context) explained just 1% of the integration or separation strategy’s variance, and 13% of assimilation adoption (Tables 6, 7).

**Table 6:** Level 2 values and acculturation strategies: unstandardized estimates and 95% CI of mixed effects linear regression for study 2 (*N* = 415).

	Acculturation strategies						Integration		Assimilation		Separation	
Fixed part
Individual values						
Personal focus	**0.185** ^*^	**[0.088, 0.283]**	**0.201** ^*^	**[0.108, 0.294]**	**−0.195** ^*^	**[−0.296, −0.095]**
Social focus	**0.389** ^*^	**[0.287, 0.490]**	**−0.146** ^*^	**[−0.244, −0.048]**	**0.241** ^*^	**[0.136, 0.346]**
Sociodemographic variables						
Gender (1 = male)	0.014	[−0.018, 0.046]	0.003	[−0.028, 0.035]	−0.003	[−0.036, 0.030]
Age	0.001	[−0.001, 0.002]	0.001	[−0.002, 0.002]	−0.001	[−0.002, 0.001]
Education	−0.013	[−0.028, 0.002]	0.001	[−0.014, 0.015]	0.007	[−0.008, 0.023]
Income	0.020	[−0.001, 0.041]	−0.004	[−0.024, 0.016]	0.009	[−0.013, 0.030]
Length of residence	−0.001	[−0.004, 0.002]	0.002	[−0.001, 0.004]	−0.003	[−0.005, 0.001]
Random part			
σ^2^	0.03	0.02	0.03
τ00 Host Country	0.01	0.01	0.01
ICC Host Country^#^	0.01	0.15	0.03
*N* Host Country	34	34	34
*R^2^*Marginal/*R^2^*Conditional	0.21/0.21	0.05/0.19	0.07/0.09

Bold figures are significant.

* *p* < 0.05.

# When replacing Host Country by the Grouping host country variable (Arab-Muslim countries, non-Arab Muslim countries, non-Muslim Asian countries, and Western countries), ICC becomes much closer to zero.

**Table 7:** Level 3 values and acculturation strategies: unstandardized estimates and 95% CI of mixed effects linear regression for study 2 (*N* = 415).

	Acculturation strategies						Integration		Assimilation		Separation	
Fixed part
Individual values						
Growth	**0.128** ^*^	**[0.011, 0.246]**	**0.187** ^*^	**[0.069, 0.305]**	**−0.233** ^*^	**[−0.356, −0.109]**
Self-protection	**0.406** ^*^	**[0.299, 0.513]**	**−0.148** ^*^	**[−0.257, −0.038]**	**0.284** ^*^	**[0.172, 0.397]**
Sociodemographic variables						
Gender (1 = men)	0.012	[−0.020, 0.043]	0.010	[−0.021, 0.042]	−0.010	[−0.044, 0.023]
Age	0.001	[−0.001, 0.003]	−0.001	[−0.002, 0.001]	0.001	[−0.001, 0.002]
Education	−0.012	[−0.027, 0.002]	0.002	[−0.012, 0.017]	0.006	[−0.009, 0.021]
Income	0.018	[−0.002, 0.038]	−0.001	[−0.020, 0.020]	0.004	[−0.018, 0.025]
Length of residence	−0.001	[−0.004, 0.001]	0.001	[−0.001, 0.004]	−0.002	[−0.005, 0.001]
Random part			
σ^2^	0.02	0.02	0.03
τ00 Host Country	0.01	0.01	0.01
ICC Host Country^#^	0.01	0.13	0.01
*N* Host Country	34	34	34
*R^2^*Marginal/*R^2^*Conditional	0.23/0.23	0.03/0.16	0.07/0.08

Bold figures are significant.

* *p* < 0.05.

# When replacing Host Country by the Grouping host country variable (Arab-Muslim countries, non-Arab Muslim countries, non-Muslim Asian countries, and Western countries), ICC becomes much closer to zero.

Supplementary Tables S4–S6 in the Supplementary material display similar analyses pertaining to the relationship between values and each of the acculturation strategy dimensions (heritage vs. host society) for interested readers.

#### Discussion

These findings were generally in line with those reported in *Study* 1, except that integration was not associated with self-transcendence and that assimilation was positively linked to self-enhancement instead of openness to change. Again, the values of conservation and self-protection (and also social-focus) were positively associated with a motivation for separation and integration, and negatively with endorsing assimilation. Beyond the support for our research hypotheses, the findings also indicated that integration, similar to assimilation was positively associated with self-enhancement, personal focus, and growth values, and that these values were negatively associated with separation. Our analyses indicated that acculturation preferences among Syrian refugees were also mainly related to motivational values, rather than to the settlement context. This was true for the separation and integration strategies; however, the assimilation strategy was more associated to context than to values among Syrian refugees. We conclude that the links between values and acculturation strategies are more similar than different across Arab immigrants and Syrian refugees and their acculturation contexts.

## General discussion

Are the acculturation strategies of immigrants and refugees affected by their individual motivational values to maintain their heritage culture and by their motivational values to engage in contact with and participation in the larger national society of their new country of residence? Drawing upon Schwartz’s (2012) *Theory of Basic Individual Values*, our study provides an initial answer to this overarching question and it extends this rarely-studied domain of psychological acculturation. Personal values define motivational goals that guide actions, and we thus proposed that they would influence immigrants’ and refugees’ inclination towards a particular acculturation strategy. In our examination of the values-acculturation strategy links, we utilized Schwartz’s (2012) refined higher values defined across four bipolar dimensions (conservation vs. openness to change, self-enhancement vs. self-transcendence, personal vs. social focus values, and growth vs. self-protection), and we employed a Euclidean distance measure (Arends-Tóth and van de Vijver, 2006) of acculturation strategies. Our study also inferred on the values-acculturation relationships across Arab immigrants (*Study 1*) and Syrian refugees (*Study 2*), shedding light on the stability and meaningfulness of the relationships across samples and settlement contexts for each sample.

Results of both *Studies 1* and *2* were supportive of most of our research hypotheses. Our findings support, in a broad sense, previous research highlighting the importance of studying individual values of immigrants who are acculturating (e.g., Phalet and Swyngedouw, 2004; Ryabichenko, 2016; Lepshokova, 2021). The findings, more precisely, showed that the integration and separation strategies shared positive associations with conservation, social focus, and self-protection values. Conservation values emphasize cohesion, stability, order, and preservation of traditions and social focus values regulate social relations and expectations of others; thus they are likely to surface in social interactions (e.g., Schwartz et al., 2012). Immigrants and refugees with such motivational values are doubtlessly likely to pursue maintenance of their heritage culture (i.e., a separation strategy), which may fulfil their assertion to belong to their familiar culture in their unfamiliar new society. However, our findings suggest also that these exact motivational values serve as a vehicle for immigrants’ and refugees’ responsiveness to their new society’s customs, rules, standards, norms and expectations. Thus, conservation and social focus values are not an obstacle for immigrants’ and refugees’ pursuance of the integration strategy. Inclination towards the integration strategy here may fulfil immigrants’ and refugees’ motivation to form further social bonds and adhere to a further set of contact and social rules dictated by the larger society.

Relocation to a new cultural context might generate anxieties for some immigrant and refugee individuals. Our findings suggest that immigrants and refugees motivated by self-protection values, which by nature are anxiety-driven and have the goal of preventing loss and protecting against threat, are likely to promote heritage culture maintenance and also likely to pursue an integration strategy. In both instances, belonging fulfil immigrants’ and refugees’ need for self-protection through the maintenance of social bonds within their heritage culture as well as the formation of new ones within the larger society. The findings across both studies indicated that while the values of conservation, self-protection, and social-focus were positively associated with endorsing separation and integration, these values were negatively associated with endorsing assimilation. Tentatively speaking, conservation, self-protection, and social-focus values, compared to other values, seem more powerful in clearly differentiating between immigrants and refugees who endorse integration or separation from those who endorse assimilation.

Differentiated from the shared values profile that integration has with separation, the integration strategy was positively associated also with self-transcendence values among immigrants (*Study 1*). Self-transcendence values emphasize concern for, and the welfare and acceptance of all, thus allowing for respect for both heritage and host society values. Integration endorsement seems to fulfil these underlying needs for self-transcendent immigrants. Research indicates that self-transcendence associates positively with a harmonious set of minority-majority interethnic relationships (e.g., Lepshokova, 2021), an outcome that integration also fulfils (e.g., Berry et al., 2022; Abu-Rayya and Brown, 2023). Hence, integration likely provides a satisfactory mechanism that actualizes the self-transcendence motivation value of some immigrants. Why was self-transcendence not associated with integration among our refugees sample (*Study 2*)? This might be attributed to the fact that refugees experience harsher realities of push factors (Berry, 1992) and that what drives them initially would be values that emphasize protection and order (i.e., conservation, social focus, and self-protection values). Self-transcendence values might drive them later in the process of acculturation and encourage an integration strategy, as is the case for immigrants.

Results of *Study 1* also showed, as expected, a positive association between the assimilation strategy with openness to change, personal focus, and growth values. The individual person is at the core of these values, much more than conservation, social focus, self-protection, and self-transcendence values which are, broadly speaking, socially driven motivations. Openness to change values emphasize independence, autonomy, change of the status quo, and readiness for new experiences/ideas, and in line with previous research (e.g., Phalet and Swyngedouw, 2004), seems to have facilitated assimilation endorsement among our immigrants sample.

Extending previous research, our findings suggest that immigrants motivated by personal focus values (that regulate personal interests’ expression and characteristics), and growth values (that free the individual from anxieties and promote their personal goals gain and self-expansion), were inclined towards the assimilation strategy. Relocation to a new cultural context might generate various (e.g., psychological, social, physical, health, economic) self-flourishing opportunities and challenges for some immigrant individuals to capitalize on. Our findings suggest that immigrants motivated by openness to change, personal focus, and growth values are likely to act upon those opportunities and challenges through the pursuance of an assimilation strategy in order to satisfy their motivations.

Results of *Study 2* replicated the associations of assimilation with personal focus and growth values. Interestingly, contrary to our research hypothesis and also to previous research that showed a positive relationship between self-enhancement values and the assimilation strategy (e.g., Ryabichenko, 2016), we found no such relationship among our Arab immigrants sample (*Study 1*). In principle, self-enhancement values emphasize values characterized by dominance, achievement, and power and would thus motivate immigrants towards the assimilation strategy. The divergence of our finding from previous research could be genuine in that it relates to the specificities of our sample; or it could be due to the new way we measured values and acculturation. We are inclined to believe that the observed null relationship is sample-specific because *Study 2* found a positive association between self-enhancement and assimilation among refugees but not between openness to change and assimilation. It might be that refugees who have been forced to escape harsh realities initially prioritize values stressing success, control and power to regain control over their life conditions. Immigrants are more relaxed in this regard and thus might probably prioritize openness to change values that become a vehicle for their assimilation, compared to self-enhancement as a motivation to assimilation among refugees. Findings of *Study 2* also indicated that integration, similar to assimilation was positively associated with self-enhancement, personal focus, and growth values, and that these values were negatively associated with separation. We are inclined to believe that self-enhancement, personal focus, and growth values are powerful in clearly differentiating between refugees who endorse assimilation or integration from those who endorse separation. Although separation and integration share positive associations with conservation, self-protection, and social-focus values, they are differentiated by the positive association of integration, and negative association of separation, with the values of self-enhancement, personal focus, and growth values. Integration is not just a group-promotion strategy among refugees but also self-promotion strategy. Refugees who seek integration are motivated by values that balance their group-interests (e.g., conservation values) and self-promotion values by looking for self-expansion and by regulating their personal interests and success in the new society.

Overall, findings of both studies indicate more similarities than differences between the value-acculturation strategy relationships across the two samples, implying that the relationships seem stable across immigrant and refugee groups with just slight differences being noticed.

Additionally, our findings demonstrated that the abovementioned relationships between values and acculturation strategies were mainly shaped by the motivational values, with a negligible role played by the settlement context of our respondents. Partialling out the value effect from the context effect in the examinations of the value-acculturation strategy links contributes uniquely to the relevant literature, we believe. Noticeably, our samples were comprised of Arab immigrants and Syrian refugees living in distinct acculturation contexts: some were acculturating in Arab Muslim countries, other were immigrants to non-Arab Muslim countries, non-Muslim Asian countries, or to Western countries. In all analyses of the associations between individual values and acculturation strategies carried out in *Study 1*, the settlement context contributed just 1–2% of the total variance of the strategies. This finding, though, should not be taken to undermine contextual effects on immigrants’ acculturation. Contextual factors like personal experiences of discrimination are well known to impact immigrants’ acculturation (e.g., Schmitz and Schmitz, 2022), and such context-unique factors were not analyzed in our study. Nevertheless, the research finding here indicated that values are more important than the particular context in shaping Arab immigrants’ acculturation strategy. *Study 2* replicated these findings in the case of integration and separation: the settlement context contributed just 1–3% of the total variance of these strategies. In the case of assimilation, however, it appears that the settlement context contributed 13–15% of the total variance, a much larger contribution than the one made by the values themselves. Refugees escape harsh realities that inevitably cause them to start restructuring a new conception of self and social belonging. To convey being capable of fitting in, they might respond to contexts that put an emphasis on assimilation of new members by claiming assimilation, or that they perceive that the host society requires them to assimilate, more than it actually does, and thus they respond by endorsing assimilation. The particularities of context-specific factors that play a role in the acculturation of our respondents in both studies were not examined.

All together, the findings of the two studies indicate that acculturation strategies are mainly related to motivational values, rather than to different settlement contexts for both immigrants and refugees, except that assimilation seems to be more associated to context than values among the refugee sample.

### Implications

Thus far, Schwartz’s (2012) *Theory of Basic Individual Values* have played a limited role in research on the acculturation strategies adopted by migrants. The current research findings are encouraging as they explain value-driven motivations underlying immigrants’ and refugees’ acculturation strategies. The findings would also potentially contribute to a refined framework of acculturation-adaptation and values-adaptation interfaces in the context of immigration. To elucidate, acculturation research has established, for instance, that the integration strategy contributes to immigrants’ wellbeing (e.g., Berry et al., 2022; Abu-Rayya et al., 2023) and intergroup harmony (Berry et al., 2022; Abu-Rayya and Brown, 2023). A separate line of research indicates that values contribute to wellbeing, generally (e.g., Grosz et al., 2021) and explain outgroup attitudes (e.g., See et al., 2020; Lepshokova, 2021). By merging of these lines of research, with the values-acculturation strategy links we established in the current study, it appears that acculturation strategies may play a mediation role in the relationships between immigrants’ and refugees’ values and adaptation outcomes. Future research may illuminate the specificities of these links and generate testable models. Based on current findings, one specific testable model would, for instance, be that immigrants’ self-transcendence values facilitate their favorability towards an integration strategy, which, in turn, improves both their adaptation and positivity towards the host society.

Our findings may also have practical implications. We propose that theorizing on the values-acculturation interface guides predictions related to immigrants’ and refugees’ willingness to integrate into the larger society of settlement. The current study suggests, for instance, that three motivational values (conservation, social focus, and self-protection) explain immigrants’ and refugees’ inclination to both separation and integration strategies. Research shows that the importance people place on individual values may be downgraded if social contexts do not provide relevant opportunities for their pursuance (e.g., Schwartz and Bardi, 1997; Lönnqvist et al., 2011). Since conservation, social focus, and self-protection values contribute to both separation and integration, any attempt to deprive immigrants and refugees of opportunities to act upon this set of values in the hope to integrate them will only harm their integration. Instead, social and personal opportunities should be provided to facilitate the pursuance of self-transcendence values, and also values of personal-focus and growth, since those values were uniquely correlated with inclination towards adopting the integration strategy, compared to endorsing separation.

Although this research did not assess psychological wellbeing, we found that the integration acculturation strategy is associated with the basic values of conservation, social focus, and self-protection, and to some extent self-transcendence, persona-focus and growth. Given the findings of other research that the integration strategy is associated with better psychological adaptation (e.g., Berry et al., 2022; Abu-Rayya et al., 2023), is it possible to promote the retention of these values (where they are already present) and their enhancement (through specific programs) in immigrant and refugee populations? Such primary prevention has been advocated, and found to be effective, in other immigrant and refugee populations (Williams and Berry, 1991). It seems worthwhile to use the findings of this research to assist in the development of such programs by focusing on the preservation and development of these basic values.

### Limitations

A few study caveats should be noted. First, although our findings are based on a relatively sizable samples of Arab immigrants and Syrian refugees acculturated to various contexts, our samples were neither representative nor random, thus generalizability of the findings must be treated with caution. Second, both of the immigrants and refugee samples comprised relatively highly educated respondents. Although we controlled for education in the analyses, the reported results might have been biased by the overall educational characteristics of the samples. Third, our approach to examine and compare the acculturation contexts of immigrants and refugees in both studies was indirect. We did not, for instance, include any acculturation-context specific measure like perceived discrimination in the analyses. Fourth, our inferences regarding similarities and differences between the immigrant and refugee samples in values and acculturation were not based on solid analyses. We analyzed each sample separately since the two samples did not acculturate to the exact host countries and we also did not include direct measures of push and pull factors in the analyses. Fifth, this study employed a cross-sectional methodology to test the links between individual values and acculturation strategies. To facilitate a more rigorous investigation of the relationships between values and acculturation, future research may utilize some of the exploratory links reported in the present study and design particular experiments to test them by manipulating the salience of values. Manipulation of values’ salience has successfully been implemented in some previous studies (e.g., Maio et al., 2009; See et al., 2020). Lastly, qualitative techniques are warranted in further research to complement our understanding of the relationships between values and acculturation preferences, as also noted by Zlobina et al. (2008).

### Conclusion

Analyses of the relationships between individual values and acculturation strategies help uncover the motivational basis underlying the adoption of acculturation strategies. Our findings made clear that the acculturation strategies are anchored in certain differential motivational values. Although slight differences emerged between the immigrants and refugees samples, the separation and integration strategies were consistently positively associated with conservation, social focus, and self-protection values and the assimilation strategy consistently associated with personal focus and growth values in both the immigrant and refugee groups. Compared to the motivational values, the settlement context *per se* played a very minor role in the acculturation strategies adopted by migrants. This pattern appeared consistent in the refugee sample as well for the separation and integration strategies; the assimilation strategy, on the other hand, appeared more associated to context than values among refugees. Further research is warranted to replicate and extend our conclusions.

## Data availability statement

The datasets presented in this study can be found in online repositories. The names of the repository/repositories and accession number(s) can be found at: https://osf.io/thgrj.

## Ethics statement

The studies involving human participants were reviewed and approved by the RBI at Doha Institute. The patients/participants provided their written informed consent to participate in this study.

## Author contributions

HA-R initiated the project. HA-R and MA provided the data. HA-R, JB, DG, and ZL conceptualized the study. DG and ZL conducted the data analyses. HA-R, JB, and ZL prepared the preliminary literature review. HA-R prepared the full article. JB contributed further to the literature review. DG, ZL, and MA provided critical revisions. All authors approved the final version of the manuscript for submission.

## Funding

Open Access funding provided by the Qatar National Library.

## Conflict of interest

The authors declare that the research was conducted in the absence of any commercial or financial relationships that could be construed as a potential conflict of interest.

## Publisher’s note

All claims expressed in this article are solely those of the authors and do not necessarily represent those of their affiliated organizations, or those of the publisher, the editors and the reviewers. Any product that may be evaluated in this article, or claim that may be made by its manufacturer, is not guaranteed or endorsed by the publisher.

## Footnotes

^1^ https://osf.io/thgrj
