# Supplementary material for: Basic values as a motivational framework relating individual values with acculturation strategies among Arab immigrants and refugees across different settlement contexts
Source: Front Psychol. 2023 Jun 5;14:1094193. doi: 10.3389/fpsyg.2023.1094193 (PMC10278764; doi:10.3389/fpsyg.2023.1094193)
Supplement: Supplementary file 1 [file Table_1.DOC]

**Basic Values as a Motivational Framework Relating Individual Basic Values with Acculturation Strategies Among Arab Immigrants and Refugees**

Supplementary Material

Table S1.

*Level 1 Values and Acculturation Dimensions:* *Unstandardized Estimates and 95% CI of Mixed Effects Linear Regression for Study 1* (*N* = 456)

|  | |  | Acculturation dimensions | | | |
| --- | --- | --- | --- | --- | --- | --- |
|  | |  | Larger society | | Heritage culture | |
| Fixed part | | |  |  |  |  |
|  | Individual values | |  |  |  |  |
|  |  | Openness to change | 0.084 | [-0.051, 0.220] | -0.134 | [-0.283, 0.015] |
|  |  | Self-enhancement | 0.117 | [-0.020, 0.253] | 0.065 | [-0.085, 0.216] |
|  |  | Conservation | 0.040 | [-0.118, 0.198] | **0.400*** | **[0.226, 0.575]** |
|  |  | Self-transcendence | **0.184*** | **[0.013, 0.355]** | 0.084 | [-0.104, 0.272] |
|  | Sociodemographic variables | |  |  |  |  |
|  |  | Gender (1 = male) | 0.028 | [-0.010, 0.066] | 0.006 | [-0.037, 0.049] |
|  |  | Age | -0.001 | [-0.003, 0.001] | 0.002 | [-0.001, 0.004] |
|  |  | Education | -0.021 | [-0.041, 0.002] | **-0.041*** | **[-0.064, -0.018]** |
|  |  | Income | 0.001 | [-0.023, 0.024] | 0.006 | [-0.019, 0.031] |
|  |  | Length of residence | 0.001 | [-0.002, 0.002] | **-0.004*** | **[-0.007, -0.002]** |
| Random part | | |  |  |  |  |
|  |  | σ^2^ | 0.04 | | 0.05 | |
|  |  | τ_00Country of Origin_ | 0.01 | | 0.01 | |
|  |  | τ_00Host Country_ | 0.01 | | 0.01 | |
|  |  | ICC _Country of Origin_ | .01 | | .06 | |
|  |  | ICC _Host Country#_ | .01 | | .03 | |
|  |  | *N* _Country of Origin_ | 18 | | 18 | |
|  |  | *N* _Host Country_ | 33 | | 33 | |
|  |  | *R^2^*_Marginal_  / *R^2^*_Conditional_ | .07 /.07 | | .13 /.20 | |

**P* < .05; # When replacing Host Country by the Grouping host country variable (Arab-Muslim countries, non-Arab Muslim countries, non-Muslim Asian countries, and Western countries), ICC becomes much closer to zero.

Table S2.

*Level 2 Values and Acculturation Dimensions:* *Unstandardized Estimates and 95% CI of Mixed Effects Linear Regression for Study 1* (*N* = 456)

|  | |  | Acculturation dimensions | | | |
| --- | --- | --- | --- | --- | --- | --- |
|  | |  | Larger society | | Heritage culture | |
| Fixed part | | |  |  |  |  |
|  | Individual values | |  |  |  |  |
|  |  | Personal focus | **0.225*** | **[0.106, 0.344]** | -0.080 | [-0.213, 0.053] |
|  |  | Social focus | **0.169*** | **[0.041, 0.298]** | **0.428*** | **[0.285, 0.572]** |
|  | Sociodemographic variables | |  |  |  |  |
|  |  | Gender (1 = male) | 0.027 | [-0.011, 0.065] | 0.004 | [-0.040, 0.047] |
|  |  | Age | -0.001 | [-0.003, 0.001] | 0.002 | [-0.001, 0.004] |
|  |  | Education | **-0.022*** | **[-0.042, -0.002]** | **-0.042*** | **[-0.065, -0.019]** |
|  |  | Income | -0.001 | [-0.023, 0.022] | 0.009 | [-0.016, 0.035] |
|  |  | Length of residence | 0.001 | [-0.002, 0.002] | **-0.004*** | **[-0.007, -0.002]** |
| Random part | | |  |  |  |  |
|  |  | σ^2^ | 0.04 | | 0.05 | |
|  |  | τ_00Country of Origin_ | 0.01 | | 0.01 | |
|  |  | τ_00Host Country_ | 0.01 | | 0.01 | |
|  |  | ICC _Country of Origin_ | .01 | | .08 | |
|  |  | ICC _Host Country#_ | .01 | | .03 | |
|  |  | *N* _Country of Origin_ | 18 | | 18 | |
|  |  | *N* _Host Country_ | 33 | | 33 | |
|  |  | *R^2^*_Marginal_  / *R^2^*_Conditional_ | .08 /.08 | | .11 /.21 | |

**P* < .05; # When replacing Host Country by the Grouping host country variable (Arab-Muslim countries, non-Arab Muslim countries, non-Muslim Asian countries, and Western countries), ICC becomes much closer to zero.

Table S3.

*Level 3 Values and Acculturation Dimensions:* *Unstandardized Estimates and 95% CI of Mixed Effects Linear Regression for Study 1* (*N* = 456)

|  | |  | Acculturation dimensions | | | |
| --- | --- | --- | --- | --- | --- | --- |
|  | |  | Larger society | | Heritage culture | |
| Fixed part | | |  |  |  |  |
|  | Individual values | |  |  |  |  |
|  |  | Growth | **0.248*** | **[0.097, 0.400]** | -0.144 | [-0.312, 0.023] |
|  |  | Self-protection | 0.108 | [-0.042, 0.258] | **0.488*** | **[0.319, 0.657]** |
|  | Sociodemographic variables | |  |  |  |  |
|  |  | Gender (1 = male) | 0.029 | [-0.008, 0.067] | -0.001 | [-0.043, 0.043] |
|  |  | Age | -0.001 | [-0.003, 0.001] | 0.002 | [-0.001, 0.004] |
|  |  | Education | **-0.022*** | **[-0.042, -0.002]** | **-0.043*** | **[-0.065, -0.020]** |
|  |  | Income | 0.001 | [-0.022, 0.023] | 0.003 | [-0.022, 0.029] |
|  |  | Length of residence | 0.001 | [-0.002, 0.002] | **-0.004*** | **[-0.007, -0.002]** |
| Random part | | |  |  |  |  |
|  |  | σ^2^ | 0.04 | | 0.05 | |
|  |  | τ_00Country of Origin_ | 0.01 | | 0.01 | |
|  |  | τ_00Host Country_ | 0.01 | | 0.01 | |
|  |  | ICC _Country of Origin_ | .01 | | .05 | |
|  |  | ICC _Host Country#_ | .01 | | .03 | |
|  |  | *N* _Country of Origin_ | 18 | | 18 | |
|  |  | *N* _Host Country_ | 33 | | 33 | |
|  |  | *R^2^*_Marginal_  / *R^2^*_Conditional_ | .07 /.07 | | .12 /.19 | |

**P* < .05; # When replacing Host Country by the Grouping host country variable (Arab-Muslim countries, non-Arab Muslim countries, non-Muslim Asian countries, and Western countries), ICC becomes much closer to zero.

Table S4.

*Level 1 Values and Acculturation Dimensions:* *Unstandardized Estimates and 95% CI of Mixed Effects Linear Regression for Study 2* (*N* = 415)

|  | |  | Acculturation dimensions | | | |
| --- | --- | --- | --- | --- | --- | --- |
|  | |  | Larger society | | Heritage culture | |
| Fixed part | | |  |  |  |  |
|  | Individual values | |  |  |  |  |
|  |  | Openness to change | 0.109 | [-0.031, 0.249] | -0.057 | [-0.210, 0.095] |
|  |  | Self-enhancement | **0.287*** | **[0.142, 0.433]** | 0.056 | [-0.102, 0.214] |
|  |  | Conservation | **0.213*** | **[0.048, 0.377]** | **0.638*** | **[0.464, 0.812]** |
|  |  | Self-transcendence | 0.068 | [-0.112, 0.248] | 0.014 | [-0.180, 0.209] |
|  | Sociodemographic variables | |  |  |  |  |
|  |  | Gender (1 = male) | 0.020 | [-0.021, 0.061] | 0.010 | [-0.035, 0.055] |
|  |  | Age | 0.001 | [-0.001, 0.003] | 0.001 | [-0.002, 0.003] |
|  |  | Education | -0.014 | [-0.034, 0.005] | -0.005 | [-0.025, 0.016] |
|  |  | Income | 0.014 | [-0.012, 0.041] | 0.027 | [-0.002, 0.055] |
|  |  | Length of residence | 0.001 | [-0.003, 0.004] | -0.003 | [-0.007, 0.001] |
| Random part | | |  |  |  |  |
|  |  | σ^2^ | 0.04 | | 0.05 | |
|  |  | τ_00Host Country_ | 0.01 | | 0.01 | |
|  |  | ICC _Host Country#_ | .08 | | .01 | |
|  |  | *N* _Host Country_ | 34 | | 34 | |
|  |  | *R^2^*_Marginal_  / *R^2^*_Conditional_ | .15 /.22 | | .21 /.21 | |

**P* < .05; # When replacing Host Country by the Grouping host country variable (Arab-Muslim countries, non-Arab Muslim countries, non-Muslim Asian countries, and Western countries), ICC becomes much closer to zero.

Table S5.

*Level 1 Values and Acculturation Dimensions:* *Unstandardized Estimates and 95% CI of Mixed Effects Linear Regression for Study 2* (*N* = 415)

|  | |  | Acculturation dimensions | | | |
| --- | --- | --- | --- | --- | --- | --- |
|  | |  | Larger society | | Heritage culture | |
| Fixed part | | |  |  |  |  |
|  | Individual values | |  |  |  |  |
|  |  | Personal focus | **0.360*** | **[0.236, 0.485]** | -0.015 | [-0.154, 0.124] |
|  |  | Social focus | **0.239*** | **[0.109, 0.370]** | **0.606*** | **[0.461, 0.750]** |
|  | Sociodemographic variables | |  |  |  |  |
|  |  | Gender (1 = male) | 0.020 | [-0.021, 0.062] | 0.015 | [-0.031, 0.060] |
|  |  | Age | 0.001 | [-0.002, 0.003] | -0.001 | [-0.003, 0.002] |
|  |  | Education | -0.017 | [-0.036, 0.002] | -0.010 | [-0.031, 0.011] |
|  |  | Income | 0.015 | [-0.012, 0.041] | 0.028 | [-0.001, 0.057] |
|  |  | Length of residence | 0.001 | [-0.003, 0.004] | -0.003 | [-0.007, 0.001] |
| Random part | | |  |  |  |  |
|  |  | σ^2^ | 0.04 | | 0.05 | |
|  |  | τ_00Host Country_ | 0.01 | | 0.01 | |
|  |  | ICC _Host Country#_ | .08 | | .01 | |
|  |  | *N* _Host Country_ | 34 | | 34 | |
|  |  | *R^2^*_Marginal_  / *R^2^*_Conditional_ | .14 /.21 | | .17 /.17 | |

**P* < .05; # When replacing Host Country by the Grouping host country variable (Arab-Muslim countries, non-Arab Muslim countries, non-Muslim Asian countries, and Western countries), ICC becomes much closer to zero.

Table S6.

*Unstandardized Estimates and 95% CI of Mixed Effects Linear Regression for Study 2* (*N* = 415)

|  | |  | Acculturation dimensions | | | |
| --- | --- | --- | --- | --- | --- | --- |
|  | |  | Larger society | | Heritage culture | |
| Fixed part | | |  |  |  |  |
|  | Individual values | |  |  |  |  |
|  |  | Growth | **0.284*** | **[0.129, 0.440]** | -0.112 | [-0.278, 0.054] |
|  |  | Self-protection | **0.264*** | **[0.120, 0.408]** | **0.664*** | **[0.513, 0.814]** |
|  | Sociodemographic variables | |  |  |  |  |
|  |  | Gender (1 = male) | 0.024 | [-0.017, 0.066] | 0.005 | [-0.040, 0.050] |
|  |  | Age | 0.001 | [-0.002, 0.003] | 0.001 | [-0.001, 0.004] |
|  |  | Education | -0.014 | [-0.034, 0.005] | -0.010 | [-0.031, 0.011] |
|  |  | Income | 0.018 | [-0.009, 0.044] | 0.021 | [-0.007, 0.050] |
|  |  | Length of residence | 0.001 | [-0.003, 0.004] | -0.003 | [-0.007, 0.001] |
| Random part | | |  |  |  |  |
|  |  | σ^2^ | 0.04 | | 0.05 | |
|  |  | τ_00Host Country_ | 0.01 | | 0.01 | |
|  |  | ICC _Host Country#_ | .08 | | .01 | |
|  |  | *N* _Host Country_ | 34 | | 34 | |
|  |  | *R^2^*_Marginal_  / *R^2^*_Conditional_ | .13 /.20 | | .20 /.20 | |

**P* < .05; # When replacing Host Country by the Grouping host country variable (Arab-Muslim countries, non-Arab Muslim countries, non-Muslim Asian countries, and Western countries), ICC becomes much closer to zero.
